# Role of exosomes in the pathogenesis, diagnosis, and treatment of central nervous system diseases

**DOI:** 10.1186/s12967-022-03493-6

**Published:** 2022-06-27

**Authors:** Yishu Fan, Zhuohui Chen, Mengqi Zhang

**Affiliations:** 1 Department of Neurology, Xiangya Hospital, Central South University, Changsha, China; 2 National Clinical Research Center for Geriatric Disorders, Xiangya Hospital, Central South University, Changsha, 410008 China

**Keywords:** Exosomes, Central nervous system diseases, CNS, Nerve injury repair, Biomarkers

## Abstract

Central nervous system (CNS) diseases, such as multiple sclerosis, Alzheimer's disease (AD), and Parkinson’s disease (PD), affect millions of people around the world. Great efforts were put in disease related research, but few breakthroughs have been made in the diagnostic and therapeutic approaches. Exosomes are cell-derived extracellular vesicles containing diverse biologically active molecules secreted by their cell of origin. These contents, including nucleic acids, proteins, lipids, amino acids, and metabolites, can be transferred between different cells, tissues, or organs, regulating various intercellular cross-organ communications and normal and pathogenic processes. Considering that cellular environment and cell state strongly impact the content and uptake efficiency of exosomes, their detection in biological fluids and content composition analysis potentially offer a multicomponent diagnostic readout of several human diseases. Recently, studies have found that aberrant secretion and content of exosomes are closely related to the pathogenesis of CNS diseases. Besides, loading natural cargoes, exosomes can deliver drugs cross the blood brain barrier, making them emerging candidates of biomarkers and therapeutics for CNS diseases. In this review, we summarize and discuss the advanced research progress of exosomes in the pathological processes of several CNS diseases in regarding with neuroinflammation, CNS repair, and pathological protein aggregation. Moreover, we propose the therapeutic strategies of applying exosomes to the diagnosis, early detection, and treatment of CNS diseases.

## Introduction

Central nervous system (CNS) disorders represent a spectrum of diseases ranging from mild neurological impairment that may have motor, sensory, visual, speech, cognitive manifestations, or a combination thereof, to comatose states and brain death [1]. At present, nearly one in six people worldwide suffer from disorders of the CNS [2]. Common CNS diseases, such as multiple sclerosis, Alzheimer's disease, and Parkinson's disease, also bring huge economy burden to the society [3, 4]. After years of investigation, CNS diseases still remain clinical challenges with limited diagnostic and therapeutic approaches [5]. Under physiological conditions, CNS is protected against potential intruders by a unique microvasculature, the blood–brain-barrier (BBB), which is composed of endothelial cells connected by tight junctions and adherent processes [6]. However, the restrictive nature of the BBB also provides an obstacle for drug delivery to the CNS [7]. Achieving sufficient drug delivery across the BBB into the brain is a key challenge for the treatment of CNS diseases, which sieves out major proportion of therapeutics [7, 8]. To overcome this problem, biologically active cargo carriers such as exosomes and nanoparticles are attracting more attentions.

Exosomes are a large component of the broader class of nanoparticles termed extracellular vehicles (EVs) (9). Small extracellular vesicles containing transferrin receptors were first reported in 1983 by Johnstone and colleagues while culturing reticulocytes [10]. These vesicles were then named as "exosome" by Johnstone in 1987 [11]. With more studies conducted, now exosomes are generally accepted as small cell-derived single-membrane vesicles (30–150 nm) released by almost all types of cells into extracellular space via fusing plasma membrane and multivesicular bodies (MVBs) [12]. Contents embedded in exosomes include nucleic acids, proteins, lipids, amino acids, metabolites, glycoconjugates, cytosolic and cell-membrane proteins [13]. Via releasing these contents to neighboring cells, as a form of paracrine signaling, and/or to distant cells, acting as a type of endocrine signaling, exosomes are able to regulate cell-to-cell communications and multiple autocrine and paracrine cellular phenotypes [14]. Exosomes recently have emerged as the most promising biomarkers for disease diagnosis and targeted drug transporter for disease treatment [15]. Compared with other synthetic drug-delivery vehicles such as liposomes and nanoparticles, exosomes have extensive and unique advantages because of their endogeneity, favorable pharmacokinetic, special immunological properties, and their ability to penetrate physiological barriers [16]. Besides, surface modification of exosomes imparts additional functionality and enable site specific drug delivery of exosomes [17]. Together, these characters make exosomes favorable in CNS diseases diagnosis and treatment.

Recent studies have revealed effective role of exosomes in the diagnosis and treatments of CNS diseases. For example, α-Synuclein in blood exosomes can help distinguish PD from multiple system atrophy [18]. Blood neuro-exosomal synaptic proteins, including GAP43, neurogranin, SNAP25, and synaptotagmin 1, act as effective biomarkers for prediction of AD 5 to 7 years before cognitive impairment [19]. Isolated exosomes could be used alone as a neurorestorative therapy in stroke and neurological injury [20]. The potential of exosomes as biomarkers and therapies for CNS disorders is being actively investigated. In this review, we systematically summarized the advanced research progress of exosomes in several common nervous system diseases. We also propose the opportunities for exosome-based approaches to neuro-restoration and targeted drug delivery in various CNS diseases.

## Biogenesis and secretion of exosomes

**Fig. 1:**
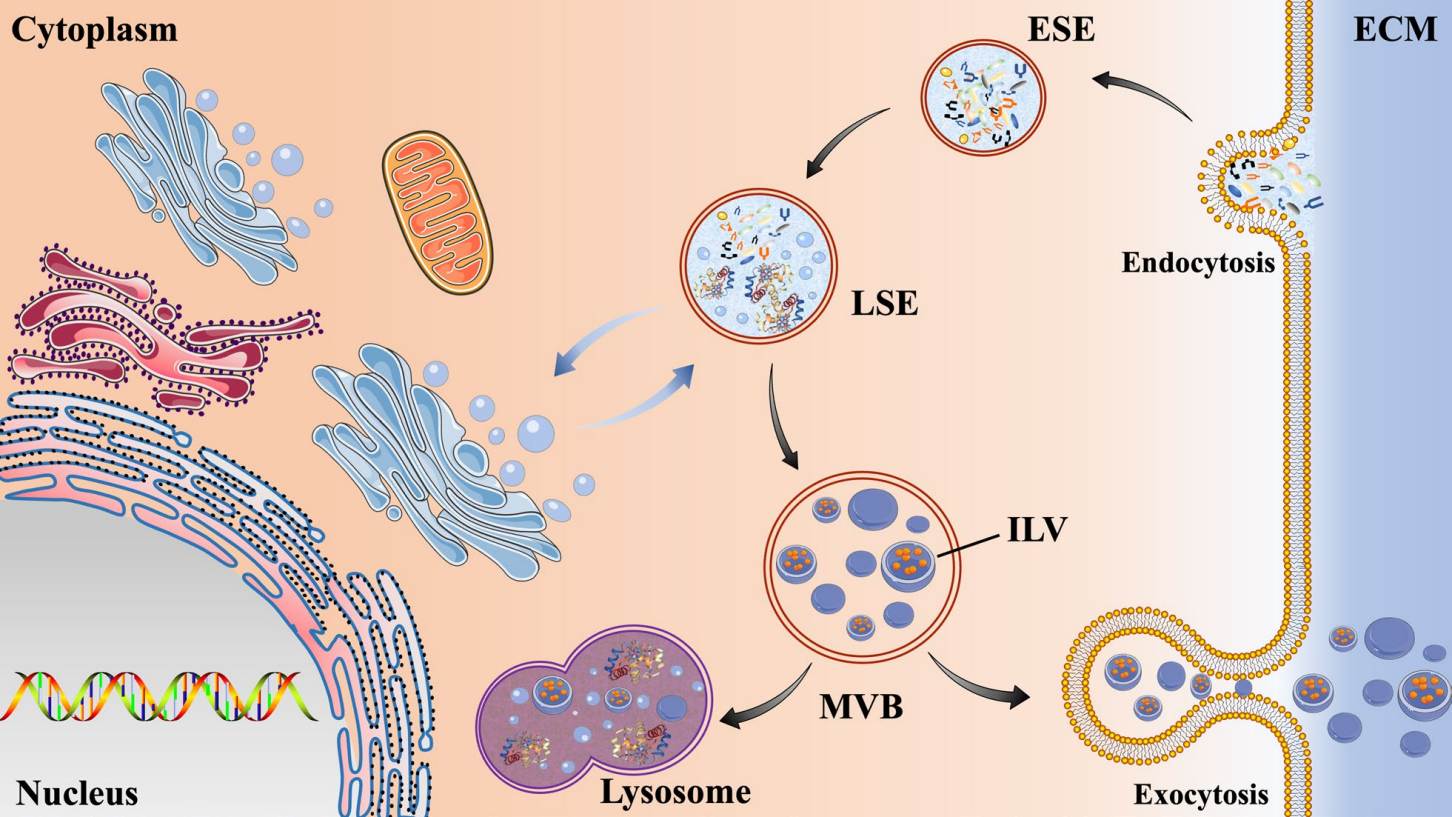
The secretion process of exosomes. Extracellular components can enter cells through endocytosis and plasma membrane depression. The vesicles formed during this process can be fused with the early sorting endosomes (ESEs) which then are transformed into the late sorting endosomes (LSEs). The second invagination in LSEs leads to the production of intraluminal vesicles (ILV). LSEs are further transformed into multivesicular bodies (MVBs), which can be degraded by fusion with lysosomes or autophagosomes, or they can be fused with plasma membrane to release ILVs as exosomes. ECM, extracellular matrix.ST

Exosomes are generated through a continuous process that involves double invagination of the plasma membrane and the formation of intracellular multivesicular bodies (MVBs) (Fig. 1) [14]. Biogenesis of exosomes starts with the de novo formation of early-sorting endosomes (ESEs). At start, cell membrane invaginates and forms a cup-shaped structure that contains cell surface proteins and extracellular components, such as soluble proteins, lipids, metabolites, small molecules, and ions [21]. Then ESEs take shape and subsequently either fuse with the endoplasmic reticulum (ER), trans-Golgi network (TGN) or a preexisting ESE. ESEs next mature into late sorting endosomes (LSEs) [22]. The second invagination in LSEs leads to the production of intraluminal vesicles (ILVs), which can further modify the load of future exosomes and allow cytoplasmic components to enter the newly formed ILVs. LSEs are further transformed into multivesicular bodies (MVBs), which can be degraded by fusion with lysosomes or autophagosomes, or they can be fused with plasma membrane to release ILVs as exosomes [23]. Exosomes are released by cells either as a reaction to specific stimuli, or under normal physiological conditions [24]. Released exosomes function as carriers of molecular information to transfer cargo molecules from parent to recipient cells and regulate cell-to-cell communications involving in physiological and pathological processes [25].

## Neuroprotective effects of exosomes

Exosomes are parts of those vesicle-producing cells. Quantity and contents of exosomes reflect the state of their cells of origin. Besides, exosome provide a mechanism by which cells can manipulate the molecular composition and function of extracellular matrix (ECM) [26]. In addition, exosomes transmit signals and molecules via a pathway of intercellular vesicle traffic, exerting local paracrine or distal systemic effects [9]. Exosome-mediated responses can be either disease promoting or restraining, depending on the composition and cell state. Furthermore, engineered exosomes can deliver diverse therapeutic payloads, including short interfering RNAs, antisense oligonucleotides, chemotherapeutic agents, and immune modulators [14]. The biological functions of exosomes span a large swath of biology, and we just focus on those aspects related to CNS diseases in this review. Exosomes released by neurons, glia and other cells in CNS contribute to the complex network of interconnected messages that underlie both the physiology and the pathology of this system [27]. Protective mechanisms of exosomes in CNS diseases which we discuss in the following include angiogenesis promotion, immune regulation, inhibiting the apoptosis of neurons and promoting the formation of myelin sheath and axon growth [28].

### Promoting angiogenesis

Exosomes derive from different cell sources, such as endothelial cells, neurons, fats, and immune cells, promote angiogenesis through different mechanisms (Table 1). Studies found that endothelial cell-derived exosomes promoted angiogenesis by secreting miR-214 which inhibited the expression of ataxia telangiectasia mutated (ATM) in other endothelial cells [29]. Exosomes secreted by adipose-derived mesenchymal stem cell (MSCs) can promote angiogenesis by transporting miR-125a to endothelial cells, inhibiting the expression of DLL4 (Delta-like ligand4) and increasing the formation of apical cells [30]. Exosomes from human umbilical cord-derived MSC can activate the Wnt4/β-catenin pathway to promote angiogenesis [31]. Exosomes from bone marrow-derived MSCs (BMSCs) can promote angiogenesis by transporting the transcription factor pSTAT3 (Signal transducer and activator of transcription (3) and activate the NF-κB pathway [32, 33]. Exosomes secreted by human term placental tissue-derived MSCs (PlaMSCs) stimulated both endothelial tube formation and migration, and enhanced angiogenesis-related gene expression [34]. Besides, an in vivo study further found that PlaMSC-exo infusion could enhance angiogenesis in a murine auricle ischemic injury model [35]. In vivo and in vitro experiments showed that miR-21-3p contained in exosomes from human umbilical cord blood (UCB) promoted the proliferation and migration of fibroblasts, and enhanced the angiogenic activities of endothelial cells [36]. In vitro and in vivo rat hindlimb ischemia model studies showed that exosomes derived from induced vascular progenitor cells promote angiogenesis [37]. Hypoxia/reoxygenation (H/R) cell culture condition stimulated human endothelial progenitor cell (EPC) to produce and release exosomes, which significantly increased fibroblast angiogenesis and the mesenchymal-endothelial transition (MEndoT) [38]. At the same time, studies showed that hypoxic-induced MSC-derived exosomes have a significantly increased composition of angiogenic substances, which can improve the treatment of ischemic diseases [39, 40]. These studies provide promising results for the clinical application of exosomes.

**Table 1:** Mechanisms of exosomes promoting angiogenesis

Source of exosomes	Intermediate molecule	Downstream pathway or molecule	Models or cells	Effect on downstream pathway	Refs.
Endothelial cell	miR-214	ATM	Endothelial cells	–	[29]
Adipose-derived MSCs	miR-125a	DLL4	Endothelial cells	–	[30]
Human umbilical cord derived MSC	NA	Wnt4/β-catenin	NA	+	[31]
Bone marrow derived MSCs	pSTAT3	NF-κB	NA	+	[32, 33]
Human term PlaMSCs	NA	Angiogenesis-related gene	In vivo murine auricle ischemic injury model	+	[34]
Human UCB	miR-21-3p	NA	Fibroblasts, endothelial cells	NA	[36]
Induced vascular progenitor cells	NA	NA	Rat hindlimb ischemia model	NA	[37]
Human EPC	NA	NA	H/R induction	NA	[38]
Hypoxic-induced MSC	NA	NA	NA	NA	[39, 40]

'NA' means no accessible data in the study.

ATM, ataxia telangiectasia mutated; MSCs, mesenchymal stem cell; DLL4, Delta-like ligand4; PlaMSCs, placental tissue-derived MSCs; UCB, umbilical cord blood; EPC, endothelial progenitor cell; H/R, hypoxia/reoxygenation

' + ' represents the promotion of downstream pathway or molecule; '–' represents the inhibition of downstream pathway or molecule;

### Neuroprotective effect

In addition to promoting angiogenesis, exosomes also exhibited neuroprotective effects to reduce the damage caused by ischemia via other mechanisms [41] (Table 2). Doeppner et al. showed that exosomes derived from MSC exhibit the same neuroprotective effects as the derived MSCs [42]. In addition, EV injection avoided the side effects related to stem cell transplantation, such as abnormal differentiation, immune rejection, and difficulty in operation [43]. Kuang et al. showed that miR-25-3p contained in exosomes produced by adipose-derived MSC exhibited neuroprotective effects by reducing neuron autophagy [44].

**Table 2:** Mechanisms of exosomal neuroprotection

Source of exosomes	Intermediate molecule	Downstream pathway or molecule	Models or cells	Effect on downstream pathway	Refs.
MSC	NA	NA	focal cerebral ischemia	NA	[42]
Adipose-derived MSCs	miR-25-3p	Autophagy	NA	–	[44]
Endothelial progenitor cell	miR-137	COX2/PGE2	Oxyhemoglobin-treated SH-SY5Y cells	NA	[45]
M2 microglia	miR-124	USP14	OGD	NA	[46]
OGD preconditioned astrocytes	miR-92b-3p	NA	OGD	NA	[49]
IPAS	circSHOC2	miR-7670-3p/SIRT1	MCAO	NA	[52]
Astrocyte	microRNA-34c	TLR7, NF-κB/MAPK	I/R injury	NA	[53]

'NA' means no accessible data in the study.

MSC, mesenchymal stem cell; OGD, oxygen–glucose deprivation; IPAS, ischemic-preconditioned astrocyte; MCAO, middle cerebral artery occlusion; I/R, ischemia/reperfusion

' + ' represents the promotion of downstream pathway or molecule; '–' represents the inhibition of downstream pathway or molecule;

In addition to MSCs, exosomes secreted by many other cells also have neuroprotective effects. miR-137 contained in endothelial progenitor cell-derived exosomes boosts the neuroprotective effect of oxyhemoglobin-treated SH-SY5Y cells partially via COX2/PGE2 pathway [45]. M2 microglia-derived exosome treatment attenuated neuronal apoptosis induced by oxygen–glucose deprivation (OGD). The underlying pathway involved exosomal miR-124 and its downstream target USP14 [46]. However, pro-inflammatory exosomes were found to accumulate in rat brains 72 h post focal cerebral ischemia. These exosomes were secreted by activated microglia with high expression of glutaminase 1 (GLS1) [47]. Applying exosome secretion inhibitor, GW4869, displayed similar anti-inflammatory effects to that of a glutaminase inhibitor, CB839. This study suggested that GLS1-mediated exosome release may play an important role in the formation of neuroinflammatory microenvironment [47].

Ischemic preconditioning (IPC) has a protective effect on ischemic brain injury. But the mechanism remains unclear [48]. Studies showed that exosomes derived from OGD preconditioned astrocytes contained an increased level of miR-92b-3p. These exosomes can be taken up by neurons and attenuated OGD-induced neuron death and apoptosis [49]. Similarly, exosomes derived from hypoxic BMSCs were found to rescue OGD-induced injury in neural cells by suppressing NLRP3 inflammasome-mediated pyroptosis and modulating microglial polarization [50, 51]. Studies had also shown that ischemic-preconditioned astrocyte-derived exosomes (IPAS-EXOs) contained high level of circSHOC2. This exosome-shuttled circSHOC2 from IPASs protected neurons from autophagy and ameliorated ischemic brain injury via the miR-7670-3p/SIRT1 axis [52].

Moreover, exosomes are crucial in protecting neurons from ischemia–reperfusion injury. Wu et al. showed that astrocyte-derived exosome-transported microRNA-34c was neuroprotective against cerebral ischemia/reperfusion injury via TLR7 and the NF-κB/MAPK pathways. [53] Mathew et al.’s research on retinal ischemia highlights the potential of MSC-EV as biomaterials for neuroprotective and regenerative therapy in retinal disorders [54]. Their results showed that administration of MSC-EVs into the vitreous humor 24 h after retinal ischemia in a rat model significantly enhanced functional recovery, and decreased neuro-inflammation and apoptosis [54]. In vitro experiments have shown that vascular endothelial cell-derived Evs reduced cell apoptosis and promote neural progenitor cell proliferation after ischemia–reperfusion [55].

## Role and application of exosomes in specific CNS diseases

Exosomes and the biomolecules they carry mediate the communication between cells in CNS which participate in the development and function of CNS [41, 56]. This is of great significance for us to study the onset and progression of CNS diseases and provide potential targets for disease diagnosis and treatments. Exosomes are involved in maintaining normal physiological functions, such as tissue repair, immune surveillance, antigen presentation, and blood coagulation processes [56–58]. However, the effects of exosomes on the body are not all beneficial, depending on their original cells and the specific molecules they contain. For instance, activated glial cell-derived exosomes carrying amyloid-β peptides and α-synuclein are pathologically linked to Alzheimer’s disease (AD) and Parkinson’s disease (PD), respectively [58]. Exosomes detected in the tumor microenvironment are found to facilitate tumorigenesis by regulating angiogenesis, immunity, and metastasis [59]. In the following text, we summarize and discuss the roles of exosomes in some specific CNS diseases, hoping to provide more information for further study and clinical application.

### Exosomes and stroke

Studies have shown that exosomes can be used as biomarkers to diagnose and stage ischemic stroke [60] (Table 3). Li et al. detected the levels of miR-422a and miR-125b-2-3p in plasma-derived exosomes of 55 patients with ischemic stroke and 25 healthy volunteers. They found that compared with the control group, the expression levels of plasma miR-422a and miR-125b-2-3p in the subacute phase group were significantly reduced, and the expression level of miR-422a in the acute phase group was significantly higher, suggesting the potential predictive value of exosomes in different periods of ischemic stroke [61].The levels of miR-233, miR-9 and miR-124 in exosomes of patients with acute ischemic stroke were higher than those in healthy people, and the expression level of these biomolecules was positively correlated with patients’ National Institutes of Health Stroke Scale (NIHS) scores [62]. Another study showed that the combination of plasma-derived miR-21-5p and miRNA-30a-5p is expected to be used as a biomarker for diagnosing and staging ischemic stroke [63]. A study conducted in rats showed that the content of rno-miR-122-5p and rno-miR-300-3p in plasma exosomes after ischemia changes significantly with time, which can be used as a potential biomarker for transient ischemic attacks [64].

**Table 3:** The role of exosomes in stroke

Source of exosomes	Intermediate molecule	Downstream pathway or molecule	Models or cells	Effect on downstream pathway	Function	Refs.
MSC	NA	IL-1β	Mice with ischemic stroke	–	Improving angiogenesis and neurogenesis	[67]
IL-4-polarized BV2 microglia cells	miRNA-26a	NA	NA	NA	Promoting angiogenesis	[68]
Endothelial cell	miR-126	NA	T2DM mice with stroke	NA	Promoting neurorestoration	[70]

'NA' means no accessible data in the study.

MSC, mesenchymal stem cells; T2DM, type 2 diabetes mellitus

'–' represents the inhibition of downstream pathway or molecule

Exosomes derived from MSCs can be used to treat ischemic stroke [65] via improving functional recovery and enhancing neurite remodeling, neurogenesis and angiogenesis [66]. MSC-exos intravenously injected into mice with ischemic stroke were shown to migrate into the brain, suppress IL-1β expression and improve angiogenesis and neurogenesis, exerting a therapeutic effect on ischemic stroke [67]. Research by Tian et al. showed that IL-4-polarized BV2 microglia cells promoted angiogenesis after ischemic stroke by secreting exosomes containing miRNA-26a [68]. Zheng et al. showed that exosomes from LPS-stimulated macrophages promoted the polarization of microglia from the harmful pro-inflammatory M1 phenotype to the beneficial anti-inflammatory M2 Phenotype, providing neuroprotection and functional improvement after ischemic stroke [69]. Venkat Poornima et al. [70] studied the neurorestorative effect of mouse brain endothelial cell exosomes on type 2 diabetes mellitus (T2DM) mice with stroke. They found that endothelial cell-derived exosomes significantly improved the neurological and cognitive functions of T2DM mice with stroke, and increased axon density, myelin density, vascular density, arterial diameter, and induced M2-macrophage polarization in the ischemic border zone [70].

In addition, engineered exosomes can be used as a drug targeting carrier in the treatment of stroke. Li et al. treated the permanent middle cerebral artery occlusion (PMCAO) stroke rat model with Edaravone (Edv)-loaded macrophage-derived exosomes. Results showed that exosomes increased the local concentration and the bioavailability of Edv. Besides, exosomes helped Edv get access to the ischemic side, reducing the death of neuronal cells and promoting the polarization of microglia from M1 to M2 [71]. Recent studies have highlighted the importance of treating secondary injuries of peripheral organs after stroke to improve overall recovery, suggesting that systemic application of MSC-derived exosomes has certain significance in improving the quality of life of ischemic stroke patients [72].

### Exosomes and gliomas

Glioma is a kind of tumor derived from neuroepithelium, accounting for 40–50% of craniocerebral tumors, which is the most common kind of intracranial tumors. Studies have shown that exosomes are closely related to the occurrence and development of gliomas [73–75] (Table 4). Glioma stem-like cells (GSCs) contribute to temozolomide (TMZ) resistance in gliomas, the mechanism of which has not yet been elucidated [76]. Yin et al.’s study showed that extracellular vesicles derived from hypoxic GSCs conferred TMZ resistance on glioblastoma by delivering miR-30b-3p, which may become a potential target for controlling chemotherapeutic resistance in tumors [77]. The increased expression of microRNA-21 (miR-21) in glioma cells is related to the immune escape probably via inhibiting paternally expressed gene 3 (PEG3) [78]. Research by Yang et al. showed that M2 bone marrow-derived macrophage (BMDM)-derived exosomes (BMDM-Exos) shuffled miR-21 to glioma cells, inhibiting their apoptosis and facilitating their invasion, proliferation and migration [78]. Besides, exosomes derived from malignant gliomas are limited in their ability to directly initiate peripheral immunosuppression [79]. Guo et al. found that hypoxia-induced glioma cells promoted the differentiation of functional MDSCs by transferring exosomal miR-29a and miR-92a to myelogenous suppressor cells (MDSCs) [80]. MDSCs play a key role in mediating the formation of an immunosuppressive environment and assisting tumors to escape the host immune surveillance [80]. Mirzaei Reza et al. found that stem-like brain tumor initiating cells (BTICs) with high resistance to radiation and chemotherapy produced and secreted exosomes containing ECM protein tenascin-C (TNC). These exosomes, once released, inhibited T cell activity and suppress the function of immune system [81]. These exosome-related studies provide new ideas for solving the problem of poor prognosis of glioblastoma.

**Table 4:** The role of exosomes in gliomas

Source of exosomes	Intermediate molecule	Downstream pathway or molecule	Models or cells	Function	Refs.
Hypoxic GSCs	miR-30b-3p	NA	NA	Conferring TMZ resistance on glioblastoma	[77]
M2 BMDM	miR-21	PEG3	NA	Promoting immune escape of glioma cells	[78]
Hypoxia-induced glioma cells	miR-29a, miR-92a	NA	NA	Promoting immune escape	[80]
Stem-like BTICs	TNC	T cell	NA	Suppressing the immune system	[81]
NA	miR-301a	Wnt / β-catenin	NA	Reducing radiosensitivity	[85]
MSC	miR-146	NA	Rat primary brain tumor model	Inhibiting the growth of glioma xenografts	[88]
WJ-MSCs	miR-124	NA	GBM	Enhancing the sensitivity to TMZ and reduce the migration	[89]

'NA' means no accessible data in the study.

GSCs, Glioma stem-like cells; TMZ, temozolomide; BMDM, bone marrow-derived macrophage; BTICs, brain tumor initiating cells; TNC, tenascin-C; MSC, mesenchymal stem cells; WJ-MSCs, Wharton's Jelly- Derived Mesenchymal Stem Cells; GBM, glioblastoma multiforme

Exosomes can also be used as biomarkers for gliomas diagnosis [73–75, 82]. Serum levels of exosomal miR-21, miR-222, and miR-124-3p of patients with high-grade glioma were significantly higher than those with low-grade glioma and control group, while the levels decreased significantly after surgery [83]. Besides, some studies exosomal miR-301a were shown to reflect the disease progression and pathology of glioma patients [84]. miR-301a was also a new target for radiotherapy resistance of glioma cells. Xiao et al. found that exosomal miR-301a in exosomes was an effective regulator of Wnt/β-catenin that can reduce radiosensitivity by targeting the tumor suppressor gene TCEAL7 [85]. Research by Huang et al. showed that the expression level of polymerase I and transcriptional release factor (PTRF) were positively correlated with associated with the degree of malignancy and poor prognosis of glioma patients [86]. Serum exosomal PTRF in GBM patients was reduced after surgery. Therefore, PTRF in serum exosomes can be used as a biomarker in the diagnosis of gliomas. It has certain advantages such as easy access to specimens, convenient testing, earlier changes, and less damage.

For disease treatment, Ning et al. showed that exosomes derived from dendritic cells can inhibit the development of gliomas and extend the lifespan of tumor-bearing mice [87]. Mark Katakowski et al. found that intra-tumoral injection of miR-146 expressed MSCs exosomes can significantly inhibit the growth of glioma xenografts in the rat primary brain tumor model [88]. Sharif s et al. found that human umbilical cord Wharton’s Jelly-Derived Mesenchymal Stem Cells (WJ-MSCs) can transfer exogenous miR-124 to glioblastoma multiforme (GBM) cells, enhancing the sensitivity of GBM cells to temozolomide and reducing the migration of GBM cells [89]. This research may provide a new way for miRNA replacement therapy of GBM. In addition, the exosome molecules are small enough to cross the blood–brain barrier as drug carriers. Studies have shown that exosomes derived from glioblastoma cells can be used to carry chemotherapeutic agents, such as paclitaxel (PTX), acting on the brain and inhibiting the growth of gliomas [90]. Jia et al. designed glioma-targeted exosomes, which can carry nanomaterials and chemical reagents to simultaneously diagnose and treat gliomas, providing a new possibility for the diagnosis and treatment of glioma [91].

### Exosomes and neurodegenerative diseases

Neurodegenerative diseases are caused by the loss of neurons or myelin sheaths, which deteriorate over time and become dysfunctional. Common neurodegenerative diseases include PD, AD, Huntington’s disease, amyotrophic lateral sclerosis, etc. Stem cell-derived exosomes produce neuroprotective effects by reducing oxidative stress [92]. Gui et al. performed microRNA analysis on exosome miRNAs in the cerebrospinal fluid (CSF) of PD and AD patients. They found that in PD-CSF, the expression of 16 exo-miRNAs was up regulated and the expression of 11 miRNAs was down regulated. RNA molecules in CSF exosomes were reliable biomarkers and were quite robust in terms of specificity and sensitivity in distinguishing PD from healthy and disease controls, such as AD [93].

#### Exosomes and Parkinson’s disease (PD)

Serum neuronal exosomes can be used to predict and distinguish PD from atypical PD. For example, exosomal α-synuclein remained stably elevated with PD progression and was positively correlated with the severity of the disease [94]. Jiang et al. analyzed serum samples from patients with different diseases, and the results showed that α-synuclein in combination with clusterin in serum neuronal exosomes predicted and differentiated PD from atypical parkinsonism [95]. In addition, miR-1, miR-19b-3p, miR-153, miR-409-3p and miR-10a-5p in the exosomes from CSF may also be used as the biomarkers for the diagnosis of PD [93]. Cellular prion protein (PrP) has been suggested to play a role in cognitive decline in PD patients. Leng et al. showed that the plasma exosomal PrP level was negatively correlated with the cognitive level in PD patients and might be a potential biomarker for PD patients who had risk of cognitive impairment [96]. Chang et al. found that exosomes secreted by α-synuclein treated microglia may be an important medium to induce PD neurodegeneration [97] Exosomes also mediate the spread of α-synuclein in PD. Guo et al. confirmed the presence of α-synuclein oligomer in microglia/macrophage derived exosomes in the CSF of PD patients, which were able to induce α-synuclein aggregation in neurons [98]. Another study showed that exosomes of human dental pulp stem cells treated with 6-hydroxydopamine (6-OHDA) can inhibit the apoptosis of dopaminergic neurons, which provided a new idea for the treatment of PD [99].

#### Exosomes and Alzheimer’s disease (AD)

The role of exosomes in AD is controversial. On the one hand, exosomes transfer toxic amyloid β and hyperphosphorylated tau protein between cells, thus inducing neuron apoptosis. However, on the other hand, exosomes can absorb brain amyloid β through microglia, and transfer neuroprotective substances between cells [100].

The study conducted by Goetzl et al. showed that with the progression of AD, the level of functionally specialized synaptic proteins in plasma neuron-derived exosome (NDE) decreased. Reductions in NDE levels of these specialized excitatory synaptic proteins may indicate the extent of cognitive loss and may reflect the severity of AD [101]. Gao et al. found that glutaminase C can regulate the activation of microglia and the release of proinflammatory exosomes, which may be closely related to the pathogenesis of AD [102]. Exosomes are available for developing biomarkers for the staging of sporadic AD. Fiandaca et al. extracted and quantified AD pathogenic proteins from neural-derived blood exosomes. Compared with healthy control group, the mean levels of exosomal total tau, P-T181-tau, P-S396-tau, and amyloid β 1–42 (Aβ1-42) in AD group were significantly higher, even 1 to 10 years before diagnosed with AD [103]. These findings indicated the ability of exosomes to predict the development of AD. In addition, research by Yang et al. showed that exosomal miR-135a and miR-384 were up-regulated while miR-193b was down-regulated in serum of AD patients. The combination of miR-135a, -193b, and -384 was proved to be better than a one for early AD diagnosis [104]. The expression of miR-342-3p in plasma exosomes of AD patients was down regulated [105]. The study by Cha et al. showed that miR-212 and miR-132 were downregulated in neural derived plasma exosomes of Alzheimer’s patients [106]. Fotuhi SN et al. found that BACE1-AS-lncRNA level in plasma and plasma-derived exosomes are significantly different between the AD subgroup and the control group, showing the possibilities of one of the biomarkers for AD [107].

Research by Lee et al. showed that exosomes secreted by adipose-derived stem cell can reduce β-amyloidosis and neuronal apoptosis in AD transgenic mouse models and enhance the growth of axons in the brain of AD patients [108]. Michael et al. found that GW4869 (an exosome secretion inhibitor) reduced the formation of amyloid plaques by preventing exosome secretion, and proved that neutral sphingomyelinase 2 (nSMase2) was a potential drug target for AD by interfering with exosome secretion [109].

### Exosomes and central nervous system injury

Studies have shown that exosomes play a role in the treatment of spinal cord injury (SCI), promoting angiogenesis and providing anti-apoptosis and anti-inflammation effects [110, 111] (Table 5). Exosomes also act as intercellular communication medium and siRNA carriers. Yu et al. used exosomes secreted by miRNA-29b-modified mesenchymal stem cells to treat rats with SCI and got promising results [112]. Jiang et al. showed that neuron-derived exosome-transmitted miR-124-3p protected traumatically injured spinal cord by suppressing the activation of neurotoxic microglia and astrocytes. The mechanism may be associated with MYH9 (the target downstream gene of miR-124-3p) and PI3K/AKT/NF-κB signaling cascades [113]. Research by Zhao et al. showed that overexpression of miR-25 by BMSCs-exo can protect the spinal cord from transient ischemia [114]. Romanelli et al. used MSC-derived exosomes to treat rat contusion SCI model. These exosomes had the same protective effects of MSC, exihibiting an anti-inflammatory and anti-scarring role [115]. Guo et al. treated SCI rats by intra-nasal administration of MSC exosomes containing phosphatase and tensin homologous siRNA. They found that exosome treatment significantly promoted axon growth and angiogenesis, reduced the proliferation of microglia and astrocytes, and promoted the functional recovery of SCI rats [116]. Xu et al. found that the recovery effect of MSC-derived exosomes on SCI was regulated by exosomal miR-21/miR-19b [117]. Studies by Li et al. showed that injection of exosomes derived from miR-133b-modified MSCs can protect neurons, promote axon regeneration, and the recovery of motor function of hind limbs in rats after SCI [118]. Huang et al. treated rats with SCI with exosomes derived from miR-126-modified MSCs. Their results showed that exosomes derived from MSCs transfected with miR-126 promoted angiogenesis and neurogenesis, inhibited apoptosis and promoted the functional recovery after SCI [119]. These results indicated that exosomes could serve as a valuable therapeutic vehicle for miR delivery to the central nervous system.

**Table 5:** The role of exosomes in SCI and TBI

Source of exosomes	Intermediate molecule	Downstream pathway or molecule	Function	Refs.
SCI				
Neuron	miR-124-3p	MYH9, PI3K/AKT/NF-κB	Suppressing the activation of neurotoxic microglia and astrocytes	[113]
MSC	miR-25	NA	Protecting the spinal cord from transient ischemia	[114]
MSC	miR-21 / miR-19b	NA	Promoting recovery	[117]
NA	miR-133b	NA	Protecting neurons, promoting axon regeneration, and the recovery of motor function	[118]
TBI				
NA	miR-124	NA	Promoting the evolution of microglia M2	[122]
Microglia	miR-124-3p	NA	Inhibiting neuronal autophagy	[123]
NA	Tau protein, phosphorylated tau protein	NA	Aggravating the motor and cognitive damage	[126]

'NA' means no accessible data in the study

MSC, mesenchymal stem cells; SCI, spinal cord injury; TBI, traumatic brain injury

Similarly, exosomes derived from MSCs can improve the neural function of brain injured rats [120], probably via promoting angiogenesis, repairing neural function, anti-inflammation and so on [121] (Table 5). Yang et al. found that exosomes rich in miR-124 promoted the evolution of microglia M2 and improved the functional recovery of hippocampal nerve after brain injury [122]. Further research by Huang et al. showed that increased miR-124-3p in microglial exosomes following traumatic brain injury (TBI) inhibited neuronal autophagy and protected against nerve injury after transferred into neurons [123]. Ni et al. investigated the role of BMSC-derived exosomes at an early stage of TBI. They found that BMSCs-exosomes served a neuroprotective function by modulating the polarization of microglia/macrophages to inhibit early neuroinflammation in TBI mice [124]. Manek, R. et al. examined the cerebrospinal fluid of patients with TBI and found that after severe TBI, the injured human brain released more exosomes, which may participate in mediating cell death and neurodegeneration after TBI [125]. TBI is also associated with an increased risk of neurodegenerative diseases. Wang et al. found that after TBI, total tau protein and phosphorylated tau protein in exosomes significantly increased. These neurotoxic proteins aggravated the motor and cognitive damage after TBI [126].

### Exosomes and peripheral nerve injury

The incidence of peripheral nerve injury (PNI) is very high, often leading to severe loss of sensory and motor function of the affected limb [127]. Autologous nerve transplantation is widely accepted as the gold standard for peripheral nerve repair, but its inherent defects greatly reduce their availability [128]. Regeneration after peripheral nerve injury is still a major challenge for researchers and clinicians. Increasing evidence show that exosomes can play a neurotherapeutic effect by mediating axon regeneration, Schwann cell activation, angiogenesis and inflammation regulation [127]. Besides, peripheral nerve injury may cause neuropathic pain. Intrathecal or peripheral administration of exosomes derived from human umbilical cord MSCs have been shown to possess antinociceptive, anti-inflammation and neurotrophic effects in rat model of nerve injury-induced neuropathic pain [129, 130]. Exosomes are becoming a promising method for the treatment of PNI. The repair of peripheral nerve injury by exosomes is mainly divided into two aspects, namely the repair of peripheral nerve injury mediated by exosome miRNAs and mRNAs, and the repair of peripheral nerve injury mediated by exosome proteins (Table 6).

**Table 6:** The role of exosomes in PNI

Source of exosomes	Intermediate molecule	Downstream pathway or molecule	Models or cells	Effect on downstream pathway	Function	Refs.
ADSC	miRNA-26b	Kpna2	Schwann cells	–	Reducing autophagy of injured SCs	[132]
BMSC	NA	VEGFA	NA	NA	Promoting the regeneration of peripheral nerves	[136]
ADSC	NA	Bcl-2 mRNA, Bax mRNA	Schwann cells	+ ,–	Reducing the apoptosis of SCs	[138]
NA	NADPH oxidase 2 complexes	NOX2-PI3K-p-Akt	NA	NA	Regulating axonal regeneration	[140]

'NA' means no accessible data in the study

PNI, peripheral nerve injury; ADSC, adipose stem cells; Kpna2, karyopherin subunit alpha 2; SC, Schwann cells; BMSC, bone marrow stromal cell

' + ' represents the promotion of downstream pathway or molecule; '–' represents the inhibition of downstream pathway or molecule

Yin et al. injected exosomes derived from adipose stem cells (ADSC-Exos) into rats with crush injury of sciatic nerve. Compared with the control group, the nerve bundle membrane was relatively complete, and the autophagy of Schwann cells in the sciatic nerve was reduced [131]. Further research showed that ADSC-Exos promoted the regeneration of the myelin sheath by moderately reducing autophagy of injured SCs. This was probably achieved by miRNA-26b contained in ADSC-Exos via downregulating karyopherin subunit alpha 2(Kpna2) [132]. Roger et al. found that exosomes derived from denervated muscles can improve the accuracy of motor neuron regeneration. [133]. Olfactory ensheathing cells (OECs) have the function of promoting nerve regeneration, but their application is limited because of the oxygen-deficient environment at the injury site [134]. Zhang et al. showed that exosomal treatment significantly promoted the survival and migration of OECs in hypoxic conditions, and effectively increased brain-derived neurotrophic factor gene expression, protein levels and secretion [135]. Zhao et al. showed that BMSC-derived exosomes can promote the regeneration of peripheral nerves and that the mechanism may involve miRNA-mediated regulation of regeneration-related genes, such as VEGFA [136]. Exosomes from human adipose-derived stem cells (ADSCs) promoted sciatic nerve regeneration via optimizing Schwann cell function [137]. Liu et al. studied the effect of ADSC-Exo on the apoptosis of Schwann cells in PNI. Their results showed that ADSC-Exo reduced the apoptosis of SCs after PNI by upregulating the anti-apoptotic Bcl-2 mRNA expression and downregulating the pro-apoptotic Bax mRNA expression [138]. Reactive oxygen species (ROS) contribute to tissue damage and remodeling mediated by inflammation after injury [139]. Research by Hervera et al. showed that ROS regulated axonal regeneration through the release of exosomal NADPH oxidase 2 complexes into injured axons, and its mechanism was related to the NOX2-PI3K-p-Akt signaling pathway [140].

### Exosomes and autoimmune disease

Exosomes play an important role in autoimmune diseases such as multiple sclerosis (MS), neuromyelitis optica (NMO), acute disseminated encephalomyelitis (ADEM), myasthenia gravis (MG) and autoimmune encephalomyelitis [141, 142]. The pathogenesis and exosome treatment strategies of these autoimmune diseases are similar. Therefore, we just take MS, which has the most relevant studies, as an example to illustrate. In the MS mouse model, curcumin encapsulated by exosomes inhibited neuronal inflammation and autoimmune responses induced by myelin oligodendrocyte glycoprotein (MOG) [143]. Kimura et al. showed that circulating exosomes suppressed the induction of regulatory T cells by let-7i. Let-7i blocked the IGF1R/TGFBR1 pathway in MS, thereby regulating the pathogenesis of MS [144]. Manna et al. analyzed circulating exosome-associated miRNAs of MS patients before and after therapy. The results showed that 14 exosomal miRNAs were significantly down-regulated, while 2 exosomal miRNAs were significantly up-regulated in IFN-β-treated relapsing–remitting MS patients. This suggested the potential of exosome-associated miRNAs as biomarkers of MS treatment response [145]. Exosomes secreted by BMSCs play therapeutic roles in many autoimmune diseases and aid in tissue repair [146]. Research by Li et al. showed that BMSC-derived exosomes attenuated inflammation and demyelination of the CNS in the experimental autoimmune encephalomyelitis (EAE) rat model. This neuro-protective effect was achieved by regulating the polarization of microglia, serving as a potential method for the treatment of autoimmune and inflammatory diseases [147].

### Exosomes and intracranial infection

Intracranial infection is caused by pathogenic microorganisms such as bacteria, viruses, parasites, mycoplasma, chlamydia, Rickettsia, mold, etc. [148, 149]. These pathogenic microorganisms circulate in the blood, cross the blood–brain barrier, invade the central nervous system, and produce a series of symptoms, including encephalitis, meningitis and brain abscess [149].

Japanese encephalitis is a clinical manifestation of brain inflammation caused by the Japanese encephalitis virus (JEV) [150]. JEV infection causes permanent nerve damage and the activation of microglia, leading to a continuous inflammatory response that eventually manifests as severe encephalitis [151]. A recent study showed that activated microglial cells released let-7a and let-7b via exosomes after JEV infection and transferred them to neurons. These exosomes, taken up by neurons, caused neuronal apoptosis by activating caspase pathways [152]. In the case of JEV infection, MSC treatment can improve the survival rate of neurons [153]. However, stem cell therapy can easily lead to blockage of small blood vessels. Therefore, MSC-derived exosomes show promising prospects for the treatment of Japanese encephalitis.

Castellani Acanthamoeba is a pathogenic microorganism that can cause granulomatous encephalitis [154]. Exosomes play a key role in this pathological process. Studies have shown that this parasite-derived exosome can induce the immune response of human THP-1 cells and the cytotoxic effect of glioma C6 cells [155]. However, the mechanism remains largely unknown. Quantitative proteomic analysis of the proteins might be helpful for the understanding of the pathogenic molecules in exosomes and the underlying mechanisms that mediate the pathogenesis of the parasite.

Exosomes also involve in the progression of meningitis. Hu et al. compared exosomal microRNAs in pulmonary tuberculosis (PTB), tuberculous meningitis (TBM), non-TB disease controls and healthy state controls. They found that 6 exosomal miRNAs (miR-20a, miR-20b, miR-26a, miR-106a, miR-191, miR-486) were differentially expressed in the TB patients. And the combination of exosomal miRNAs and electronic health records (EHRs) used to diagnose TBM and PTB has a high diagnostic efficiency [156].

## Conclusions and prospects

In this review, we first summarize the potential mechanisms of neuroprotective effects of exosomes. Then we discussed the role and application of exosomes in several CNS diseases. As we discussed above, exosomes participate in the onset and progression of a variety of neurological diseases. These exosome-mediated responses can be either disease promoting or restraining, depending on their contents and the intrinsic properties of diseases. Besides, the properties of exosomes in regulating complex intracellular pathways have advanced their potential as therapeutic targets and biomarkers for early diagnosis of diseases [14]. In addition, exosomes can be engineered to deliver diverse therapeutic payloads to a desired target. Ongoing technological and experimental advances enhance our ability to harness their therapeutic and diagnostic potential. There is an increasing number of clinical trials investigating the clinical application of exosomes. Though at present, there is no finished or published clinical trials regarding the role of exosomes in the treatment of CNS diseases, several undergoing clinical trials and studies using exosomes as biomarkers are worthy of notice. For example, s clinical study performed by researchers from Isfahan University of Medical Sciences aimed to assay the effects of MSC derived exosomes on improving the disability of patients with acute ischemic stroke (ClinicalTrials.gov Identifier: NCT03384433). Another study performed by researchers from China Medical University Hospital explored the role of acupuncture-induced exosomes in the treatment of post-stroke dementia (ClinicalTrials.gov Identifier: NCT05326724). In addition, study conducted by researchers from Neurological Associates of West Los Angeles evaluated the safety and efficacy of exosome together with concurrent transcranial ultrasound in treating patients with refractory, treatment resistant depression, anxiety, and neurodegenerative dementia (ClinicalTrials.gov Identifier: NCT04202770). Researchers from Ruijin Hospital, Shanghai, China evaluated the safety and efficacy of allogenic adipose MSCs derived exosomes in patients with Alzheimer's disease (ClinicalTrials.gov Identifier: NCT04388982). Other studies are also being conducted to explore the possibility of exosomes as new biomarkers of stroke (ClinicalTrials.gov Identifier: NCT05370105), TBI (ClinicalTrials.gov Identifier: NCT04928534), intracerebral hemorrhage (ClinicalTrials.gov Identifier: NCT05035134) and PD (ClinicalTrials.gov Identifier: NCT01860118).

Despite great progress in exosome-based disease-related studies, there are still some unanswered questions that need to be addressed in the future research. For example, the exact content sorting and secretory regulation mechanisms of exosomes remain largely unknown. Besides, to better distinguish exosomes from other extracellular particles, advanced selection and isolation techniques are required for exosome separation and quantification. In addition, exosomal surface modification and therapeutic cargo loading to enable their specific cell-targeted delivery and treatment present new challenges. Most importantly, application of exosomes in the diagnosis and treatment CNS diseases and the underlying regulating mechanisms need more support evidence, especially the biomarkers for exosome isolation, the dosage, measurement standards, and administration routes of exosomes for disease treatment. Side effects, immunogenicity effects and the heterogeneity of exosomes should also be taken into consideration. Despite the existing obstacles, using exosomes as potential biomarkers and therapeutics is attractive in CNS disease, which is worthy of more investigation in the future.

## Data Availability

Not applicable.
